# Acetyl α-d-2,3,4-tri­acetyl­lyxo­pyran­oside

**DOI:** 10.1107/S2414314625000161

**Published:** 2025-01-10

**Authors:** Shawn Culver, Jonathan S. Rhoad

**Affiliations:** a Missouri Western State University, 4525 Downs Dr Saint Joseph MO 64507 USA; University of Aberdeen, United Kingdom

**Keywords:** crystal structure, carbohydrate, anomeric effect, chair conformation

## Abstract

The structure of the title compound, C_13_H_18_O_9_, has monoclinic (*P*2_1_) symmetry. It is of inter­est with respect to stereochemistry and the anomeric effect. Two acetyl subsituents adopt equatorial orientations and two are axial. The extended structure displays C—H⋯O hydrogen bonding.

## Abstract 2

In the title compound, which is of inter­est with respect to stereochemistry and the anomeric effect, two acetyl substituents adopt equatorial orientations and two are axial. The extended structure displays C—H⋯O hydrogen bonding.

## Graphical abstract

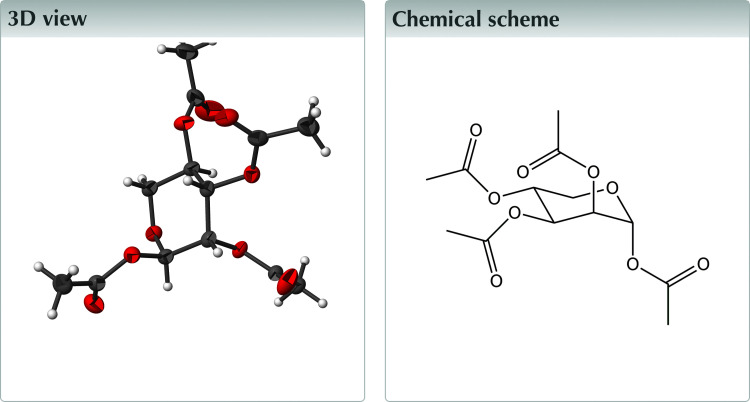

## Structure description

The anomeric effect is of inter­est to aid our understanding of conformational preferences and stereocontrol of reaction of carbohydrates and carbohydrate-like mol­ecules (Juaristi, 2024 ▸; Alabugin *et al.*, 2021 ▸). Our inter­est is in carbohydrate and carbohydrate analog ring conformations, leading to the synthesis of common carbohydrate derivatives. Recent methods have been used to try to evaluate the energy of the anomeric and related effects (Custodio Castro *et al.* 2024 ▸; Matamoros *et al.*, 2024 ▸) using complex techniques to deconvolute steric effects from electronic effects.

**Figure 1:**
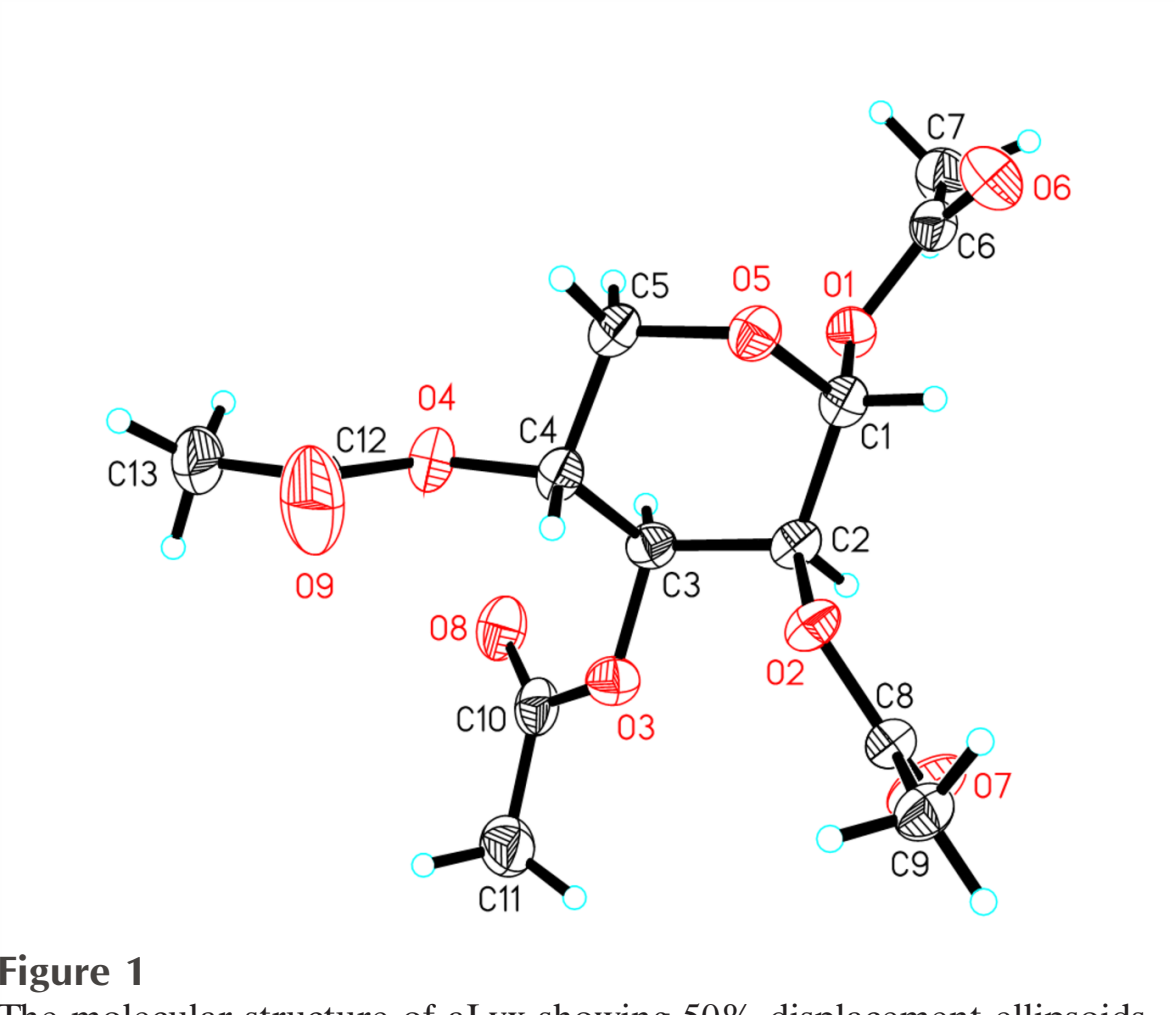
The mol­ecular structure of aLyx showing 50% displacement ellipsoids.

The crystal structure of the title compound, C_13_H_18_O_9_ (aLyx) (Fig. 1 ▸), is of inter­est because the two chair conformations each have two acetate groups in axial orientations and two acetate groups equatorial, with the acetate groups at positions 2 and 3 *cis*, so that in the chair conformations, they are always *gauche*. This means that the total energy of the steric inter­actions for each chair conformation is equal, so any difference in energy is due to the electronic inter­action at the acetal group. In the solid state, aLyx is in the ^4^*C*_1_ conformation, with Cremer & Pople (1975 ▸) puckering parameters of φ = 263 (3)°, θ = 4.3 (2)° and *Q* = 0.543 (2) Å. Since θ is close to 0°, it is in a nearly perfect chair conformation, while the *Q* parameter is lower than average, so the chair is a little flattened. The flattening is to be expected with two acetate groups in the axial position, as flattening the ring decreases *gauche* inter­actions of axial groups with the ring. The acetate substituent at the anomeric (C1) position is axial, indicating the influence of the anomeric effect. The key torsion angles are O1—C1—C2—O2 = 169.73 (14)° and O3—C3—C4—O4 = −71.1 (2)°. The configurations of the stereogenic centers are C1 *R*, C2 *S*, C3 *R* and C4 *R*, as expected for the lyxose starting material. This structure will be the starting point for calculations to qu­antify the anomeric effect in this sterically balanced mol­ecule. In the crystal, weak C—H⋯O inter­actions (Table 1 ▸) link the mol­ecules.

## Synthesis and crystallization

100 mg (6.7 mmol) of lyxose and 10 mg of sodium acetate were dissolved in approximately 2 ml of acetic anhydride. The solution was heated to reflux for 2 h. After cooling the reaction mixture to room temperature, the solution was poured over crushed ice. After the ice melted, the resulting oil was separated from the water and dissolved in minimal boiling ethanol. A few grains of activated charcoal were added to the ethanol and the solution was boiled as before. This was then passed through a cotton filter and eluted through a silica gel column with a 80:20 hexane-to-di­chloro­methane mobile phase. Upon evaporation, fine crystals were formed. The crystals were dissolved in a minimal amount of ether and allowed to evaporate overnight to form rectangular parallelepipeds of aLyx.

## Refinement

Crystal data, data collection and structure refinement details are summarized in Table 2 ▸.

## Supplementary Material

Crystal structure: contains datablock(s) I. DOI: 10.1107/S2414314625000161/hb4504sup1.cif

Structure factors: contains datablock(s) I. DOI: 10.1107/S2414314625000161/hb4504Isup2.hkl

Supporting information file. DOI: 10.1107/S2414314625000161/hb4504Isup3.cdx

CCDC reference: 2415464

Additional supporting information:  crystallographic information; 3D view; checkCIF report

## Figures and Tables

**Table 1:** Hydrogen-bond geometry (Å, °)

*D*—H⋯*A*	*D*—H	H⋯*A*	*D*⋯*A*	*D*—H⋯*A*
C1—H1⋯O8^i^	0.97 (3)	2.47 (3)	3.198 (3)	132 (2)
C5—H5*B*⋯O7^i^	0.94 (3)	2.63 (3)	3.528 (3)	160 (2)
C9—H9*C*⋯O5^ii^	0.97 (3)	2.57 (3)	3.434 (3)	149 (3)
C11—H11*C*⋯O6^iii^	1.07 (4)	2.39 (4)	3.331 (3)	145 (3)
C13—H13*C*⋯O6^iii^	0.92 (5)	2.66 (5)	3.554 (4)	162 (4)

Symmetry codes: (i) 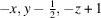; (ii) 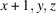; (iii) 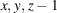.

**Table 2:** Experimental details

Crystal data	
Chemical formula	C_13_H_18_O_9_
*M*_r_	318.27
Crystal system, space group	Monoclinic, *P*2_1_
Temperature (K)	200
*a*, *b*, *c* (Å)	8.1174 (3), 9.5597 (4), 10.2580 (4)
β (°)	109.7341 (14)
*V* (Å^3^)	749.27 (5)
*Z*	2
Radiation type	Cu *K*α
μ (mm^−1^)	1.05
Crystal size (mm)	0.20 × 0.08 × 0.05

Data collection	
Diffractometer	Bruker APEXII CCD
Absorption correction	Multi-scan (*SADABS*; Krause *et al.*, 2015 ▸)
*T*_min_, *T*_max_	0.650, 0.753
No. of measured, independent and observed [*I* > 2σ(*I*)] reflections	5524, 2107, 2081
*R*_int_	0.025
(sin θ/λ)_max_ (Å^−1^)	0.610

Refinement	
*R*[*F*^2^ > 2σ(*F*^2^)], *wR*(*F*^2^), *S*	0.032, 0.076, 1.11
No. of reflections	2107
No. of parameters	270
No. of restraints	1
H-atom treatment	Only H-atom displacement parameters refined
Δρ_max_, Δρ_min_ (e Å^−3^)	0.30, −0.25
Absolute structure	Flack *x* determined using 630 quotients [(*I*^+^)-(*I*^-^)]/[(*I*^+^)+(*I*^-^)] (Parsons *et al.*, 2013 ▸)
Absolute structure parameter	0.08 (7)

Computer programs: *APEX2* (Bruker, 2017 ▸), *SAINT* (Bruker, 2014 ▸), *SHELXS97* and *SHELXTL* (Sheldrick 2008 ▸) and *SHELXL2019/1* (Sheldrick, 2015 ▸).

## full crystallographic data

### Acetyl α-D-2,3,4-triacetyllyxopyranoside Crystal data

**Table:**

C_13_H_18_O_9_	*F*(000) = 336
*M**_r_* = 318.27	*D*_x_ = 1.411 Mg m^−^^3^
Monoclinic, *P*2_1_	Cu *K*α radiation, λ = 1.54178 Å
*a* = 8.1174 (3) Å	Cell parameters from 5073 reflections
*b* = 9.5597 (4) Å	θ = 6.5–70.0°
*c* = 10.2580 (4) Å	µ = 1.05 mm^−^^1^
β = 109.7341 (14)°	*T* = 200 K
*V* = 749.27 (5) Å^3^	Rectangular parallelepiped, colourless
*Z* = 2	0.20 × 0.08 × 0.05 mm

### Acetyl α-D-2,3,4-triacetyllyxopyranoside Data collection

**Table:**

Bruker APEXII CCD diffractometer	2081 reflections with *I* > 2σ(*I*)
φ and ω scans	*R*_int_ = 0.025
Absorption correction: multi-scan (SADABS; Krause *et al.*, 2015)	θ_max_ = 70.1°, θ_min_ = 6.5°
*T*_min_ = 0.650, *T*_max_ = 0.753	*h* = −9→8
5524 measured reflections	*k* = −11→8
2107 independent reflections	*l* = −12→12

### Acetyl α-D-2,3,4-triacetyllyxopyranoside Refinement

**Table:**

Refinement on *F*^2^	Only H-atom displacement parameters refined
Least-squares matrix: full	*w* = 1/[σ^2^(*F*_o_^2^) + (0.0444*P*)^2^ + 0.0979*P*] where *P* = (*F*_o_^2^ + 2*F*_c_^2^)/3
*R*[*F*^2^ > 2σ(*F*^2^)] = 0.032	(Δ/σ)_max_ < 0.001
*wR*(*F*^2^) = 0.076	Δρ_max_ = 0.30 e Å^−^^3^
*S* = 1.11	Δρ_min_ = −0.24 e Å^−^^3^
2107 reflections	Extinction correction: *SHELXL2019/1* (Sheldrick, 2015), Fc^*^=kFc[1+0.001xFc^2^λ^3^/sin(2θ)]^-1/4^
270 parameters	Extinction coefficient: 0.028 (3)
1 restraint	Absolute structure: Flack *x* determined using 630 quotients [(*I*^+^)-(*I*^-^)]/[(*I*^+^)+(*I*^-^)] (Parsons *et al.*, 2013)
Hydrogen site location: difference Fourier map	Absolute structure parameter: 0.08 (7)

### Acetyl α-D-2,3,4-triacetyllyxopyranoside Special details

**Table:**

Geometry. All esds (except the esd in the dihedral angle between two l.s. planes) are estimated using the full covariance matrix. The cell esds are taken into account individually in the estimation of esds in distances, angles and torsion angles; correlations between esds in cell parameters are only used when they are defined by crystal symmetry. An approximate (isotropic) treatment of cell esds is used for estimating esds involving l.s. planes.
Refinement. The ring and methyl hydrogen atoms were initially located in difference-Fourier maps and refined as individual isotropic atoms.

### Acetyl α-D-2,3,4-triacetyllyxopyranoside Fractional atomic coordinates and isotropic or equivalent isotropic displacement parameters (Å^2^)

**Table:**

	*x*	*y*	*z*	*U*_iso_*/*U*_eq_	
O1	−0.12797 (18)	0.61079 (17)	0.65441 (14)	0.0265 (4)	
O2	0.18123 (17)	0.40848 (17)	0.55297 (15)	0.0261 (4)	
O3	0.05956 (18)	0.56065 (17)	0.31122 (15)	0.0278 (4)	
O4	−0.2996 (2)	0.45517 (19)	0.21466 (16)	0.0335 (4)	
O5	−0.14941 (18)	0.37458 (16)	0.58585 (15)	0.0267 (4)	
O6	−0.1547 (2)	0.5135 (2)	0.84634 (17)	0.0396 (4)	
O7	0.4179 (2)	0.5469 (2)	0.5983 (2)	0.0458 (5)	
O8	−0.1219 (2)	0.7203 (2)	0.17276 (16)	0.0374 (4)	
O9	−0.2878 (4)	0.2352 (3)	0.1434 (2)	0.0698 (7)	
C1	−0.0355 (3)	0.4874 (2)	0.6386 (2)	0.0249 (5)	
H1	0.038 (3)	0.457 (3)	0.730 (3)	0.025 (6)*	
C2	0.0676 (2)	0.5270 (2)	0.5439 (2)	0.0237 (4)	
H2	0.138 (3)	0.606 (3)	0.571 (3)	0.020 (5)*	
C3	−0.0509 (3)	0.5479 (2)	0.3942 (2)	0.0244 (4)	
H3	−0.117 (3)	0.631 (3)	0.386 (2)	0.018 (5)*	
C4	−0.1710 (3)	0.4228 (3)	0.3476 (2)	0.0260 (5)	
H4	−0.103 (3)	0.344 (3)	0.343 (2)	0.020 (6)*	
C5	−0.2688 (3)	0.3961 (3)	0.4481 (2)	0.0273 (5)	
H5A	−0.342 (3)	0.470 (3)	0.446 (2)	0.017 (6)*	
H5B	−0.336 (3)	0.315 (3)	0.427 (3)	0.021 (6)*	
C6	−0.1753 (3)	0.6122 (3)	0.7703 (2)	0.0285 (5)	
C7	−0.2522 (4)	0.7496 (3)	0.7883 (3)	0.0381 (6)	
H7A	−0.211 (2)	0.7748 (15)	0.880 (5)	0.075 (12)*	
H7B	−0.232 (7)	0.826 (6)	0.730 (6)	0.093 (15)*	
H7C	−0.369 (8)	0.738 (7)	0.761 (6)	0.104 (18)*	
C8	0.3551 (3)	0.4327 (3)	0.5903 (2)	0.0267 (5)	
C9	0.4529 (3)	0.2978 (3)	0.6181 (2)	0.0305 (5)	
H9A	0.414 (4)	0.237 (4)	0.535 (4)	0.050 (9)*	
H9B	0.428 (4)	0.251 (4)	0.691 (3)	0.034 (7)*	
H9C	0.579 (4)	0.312 (4)	0.648 (3)	0.045 (8)*	
C10	0.0097 (3)	0.6518 (3)	0.2030 (2)	0.0301 (5)	
C11	0.1387 (4)	0.6508 (4)	0.1280 (3)	0.0423 (6)	
H11A	0.246 (5)	0.623 (5)	0.185 (4)	0.051 (9)*	
H11B	0.141 (7)	0.742 (7)	0.088 (6)	0.092 (15)*	
H11C	0.090 (5)	0.577 (5)	0.045 (4)	0.060 (10)*	
C12	−0.3453 (3)	0.3515 (3)	0.1204 (2)	0.0394 (6)	
C13	−0.4761 (4)	0.4017 (5)	−0.0120 (3)	0.0540 (9)	
H13A	−0.544 (7)	0.482 (7)	0.009 (5)	0.092 (16)*	
H13B	−0.546 (6)	0.333 (6)	−0.055 (5)	0.074 (13)*	
H13C	−0.410 (6)	0.421 (6)	−0.067 (5)	0.074 (12)*	

### Acetyl α-D-2,3,4-triacetyllyxopyranoside Atomic displacement parameters (Å^2^)

**Table:**

	*U*^11^	*U*^22^	*U*^33^	*U*^12^	*U*^13^	*U*^23^
O1	0.0271 (7)	0.0251 (8)	0.0268 (6)	0.0027 (6)	0.0085 (5)	−0.0005 (6)
O2	0.0187 (7)	0.0230 (8)	0.0344 (7)	0.0007 (6)	0.0060 (5)	−0.0024 (6)
O3	0.0275 (7)	0.0268 (8)	0.0305 (7)	0.0011 (6)	0.0117 (6)	0.0024 (6)
O4	0.0309 (7)	0.0349 (10)	0.0273 (7)	−0.0029 (7)	0.0000 (6)	−0.0030 (7)
O5	0.0260 (7)	0.0231 (8)	0.0294 (7)	−0.0025 (6)	0.0073 (6)	0.0005 (6)
O6	0.0494 (9)	0.0388 (11)	0.0336 (8)	0.0015 (8)	0.0179 (7)	0.0031 (8)
O7	0.0252 (8)	0.0303 (10)	0.0781 (12)	−0.0055 (7)	0.0126 (8)	−0.0029 (10)
O8	0.0374 (9)	0.0336 (10)	0.0333 (8)	0.0012 (8)	0.0015 (6)	0.0044 (7)
O9	0.0903 (17)	0.0519 (16)	0.0528 (12)	0.0004 (14)	0.0051 (11)	−0.0247 (12)
C1	0.0236 (9)	0.0231 (11)	0.0262 (9)	0.0010 (8)	0.0061 (7)	−0.0016 (8)
C2	0.0208 (9)	0.0187 (11)	0.0306 (10)	0.0001 (8)	0.0071 (7)	−0.0019 (9)
C3	0.0228 (9)	0.0224 (11)	0.0282 (9)	0.0017 (9)	0.0090 (8)	−0.0006 (9)
C4	0.0227 (9)	0.0254 (11)	0.0265 (10)	0.0005 (9)	0.0038 (7)	−0.0025 (9)
C5	0.0222 (10)	0.0260 (12)	0.0310 (10)	−0.0018 (10)	0.0056 (8)	−0.0024 (9)
C6	0.0267 (10)	0.0316 (13)	0.0263 (9)	−0.0029 (9)	0.0075 (7)	−0.0049 (9)
C7	0.0417 (14)	0.0378 (15)	0.0361 (12)	0.0055 (11)	0.0146 (10)	−0.0067 (11)
C8	0.0212 (9)	0.0320 (12)	0.0262 (9)	−0.0009 (9)	0.0071 (7)	−0.0011 (9)
C9	0.0234 (10)	0.0325 (14)	0.0334 (11)	0.0029 (9)	0.0067 (8)	0.0030 (10)
C10	0.0326 (11)	0.0274 (12)	0.0259 (10)	−0.0081 (10)	0.0042 (8)	−0.0036 (9)
C11	0.0424 (14)	0.0515 (17)	0.0327 (12)	−0.0114 (12)	0.0124 (10)	0.0015 (12)
C12	0.0392 (12)	0.0507 (18)	0.0303 (11)	−0.0095 (12)	0.0144 (9)	−0.0103 (11)
C13	0.0473 (15)	0.084 (3)	0.0263 (11)	−0.0197 (18)	0.0065 (10)	−0.0040 (15)

### Acetyl α-D-2,3,4-triacetyllyxopyranoside Geometric parameters (Å, º)

**Table:**

O1—C6	1.367 (3)	C4—C5	1.520 (3)
O1—C1	1.437 (3)	C4—H4	0.95 (3)
O2—C8	1.352 (3)	C5—H5A	0.92 (3)
O2—C2	1.444 (2)	C5—H5B	0.94 (3)
O3—C10	1.361 (3)	C6—C7	1.493 (4)
O3—C3	1.435 (2)	C7—H7A	0.91 (5)
O4—C12	1.346 (3)	C7—H7B	0.99 (6)
O4—C4	1.443 (2)	C7—H7C	0.90 (6)
O5—C1	1.405 (3)	C8—C9	1.491 (3)
O5—C5	1.433 (2)	C9—H9A	0.99 (4)
O6—C6	1.199 (3)	C9—H9B	0.95 (3)
O7—C8	1.196 (3)	C9—H9C	0.97 (3)
O8—C10	1.201 (3)	C10—C11	1.494 (3)
O9—C12	1.198 (4)	C11—H11A	0.91 (4)
C1—C2	1.528 (3)	C11—H11B	0.96 (7)
C1—H1	0.97 (3)	C11—H11C	1.07 (4)
C2—C3	1.525 (3)	C12—C13	1.493 (4)
C2—H2	0.94 (3)	C13—H13A	1.01 (6)
C3—C4	1.515 (3)	C13—H13B	0.89 (6)
C3—H3	0.94 (3)	C13—H13C	0.92 (5)
			
C6—O1—C1	114.64 (16)	O6—C6—C7	125.7 (2)
C8—O2—C2	117.83 (18)	O1—C6—C7	111.6 (2)
C10—O3—C3	117.73 (17)	C6—C7—H7A	109.7
C12—O4—C4	117.2 (2)	C6—C7—H7B	114 (3)
C1—O5—C5	114.06 (16)	H7A—C7—H7B	110.1
O5—C1—O1	111.88 (15)	C6—C7—H7C	107 (4)
O5—C1—C2	112.09 (17)	H7A—C7—H7C	109.4
O1—C1—C2	106.69 (17)	H7B—C7—H7C	106 (5)
O5—C1—H1	104.6 (17)	O7—C8—O2	123.8 (2)
O1—C1—H1	108.5 (16)	O7—C8—C9	126.10 (19)
C2—C1—H1	113.1 (15)	O2—C8—C9	110.1 (2)
O2—C2—C3	109.81 (16)	C8—C9—H9A	110 (2)
O2—C2—C1	103.86 (16)	C8—C9—H9B	109 (2)
C3—C2—C1	112.22 (16)	H9A—C9—H9B	108 (3)
O2—C2—H2	107.6 (16)	C8—C9—H9C	112 (2)
C3—C2—H2	108.1 (15)	H9A—C9—H9C	110 (3)
C1—C2—H2	115.0 (15)	H9B—C9—H9C	107 (3)
O3—C3—C4	110.17 (17)	O8—C10—O3	123.6 (2)
O3—C3—C2	107.51 (15)	O8—C10—C11	126.0 (2)
C4—C3—C2	109.53 (17)	O3—C10—C11	110.4 (2)
O3—C3—H3	109.2 (15)	C10—C11—H11A	111 (2)
C4—C3—H3	110.3 (15)	C10—C11—H11B	109 (3)
C2—C3—H3	110.1 (14)	H11A—C11—H11B	113 (4)
O4—C4—C3	108.18 (18)	C10—C11—H11C	106 (2)
O4—C4—C5	107.47 (16)	H11A—C11—H11C	109 (3)
C3—C4—C5	110.29 (17)	H11B—C11—H11C	108 (4)
O4—C4—H4	111.8 (15)	O9—C12—O4	123.2 (2)
C3—C4—H4	108.6 (15)	O9—C12—C13	126.0 (3)
C5—C4—H4	110.5 (15)	O4—C12—C13	110.8 (3)
O5—C5—C4	111.03 (16)	C12—C13—H13A	109 (3)
O5—C5—H5A	110.3 (15)	C12—C13—H13B	111 (3)
C4—C5—H5A	109.7 (15)	H13A—C13—H13B	112 (4)
O5—C5—H5B	105.0 (15)	C12—C13—H13C	104 (3)
C4—C5—H5B	112.4 (15)	H13A—C13—H13C	117 (5)
H5A—C5—H5B	108 (2)	H13B—C13—H13C	104 (4)
O6—C6—O1	122.7 (2)		
			
C5—O5—C1—O1	64.6 (2)	C12—O4—C4—C5	−99.9 (2)
C5—O5—C1—C2	−55.2 (2)	O3—C3—C4—O4	−71.1 (2)
C6—O1—C1—O5	79.4 (2)	C2—C3—C4—O4	170.86 (16)
C6—O1—C1—C2	−157.64 (16)	O3—C3—C4—C5	171.64 (17)
C8—O2—C2—C3	114.69 (18)	C2—C3—C4—C5	53.6 (2)
C8—O2—C2—C1	−125.12 (17)	C1—O5—C5—C4	58.9 (2)
O5—C1—C2—O2	−67.48 (19)	O4—C4—C5—O5	−175.16 (18)
O1—C1—C2—O2	169.73 (14)	C3—C4—C5—O5	−57.5 (2)
O5—C1—C2—C3	51.1 (2)	C1—O1—C6—O6	−6.1 (3)
O1—C1—C2—C3	−71.7 (2)	C1—O1—C6—C7	173.50 (18)
C10—O3—C3—C4	97.3 (2)	C2—O2—C8—O7	−10.3 (3)
C10—O3—C3—C2	−143.44 (18)	C2—O2—C8—C9	170.07 (16)
O2—C2—C3—O3	−55.3 (2)	C3—O3—C10—O8	−0.8 (3)
C1—C2—C3—O3	−170.28 (18)	C3—O3—C10—C11	−179.63 (19)
O2—C2—C3—C4	64.4 (2)	C4—O4—C12—O9	1.9 (4)
C1—C2—C3—C4	−50.6 (2)	C4—O4—C12—C13	−178.94 (19)
C12—O4—C4—C3	141.0 (2)		

### Acetyl α-D-2,3,4-triacetyllyxopyranoside Hydrogen-bond geometry (Å, º)

**Table:**

*D*—H···*A*	*D*—H	H···*A*	*D*···*A*	*D*—H···*A*
C1—H1···O8^i^	0.97 (3)	2.47 (3)	3.198 (3)	132 (2)
C5—H5*B*···O7^i^	0.94 (3)	2.63 (3)	3.528 (3)	160 (2)
C9—H9*C*···O5^ii^	0.97 (3)	2.57 (3)	3.434 (3)	149 (3)
C11—H11*C*···O6^iii^	1.07 (4)	2.39 (4)	3.331 (3)	145 (3)
C13—H13*C*···O6^iii^	0.92 (5)	2.66 (5)	3.554 (4)	162 (4)

Symmetry codes: (i) −*x*, *y*−1/2, −*z*+1; (ii) *x*+1, *y*, *z*; (iii) *x*, *y*, *z*−1.
